# Pathways of Care for Patients Who Undergo Primary Percutaneous Coronary Intervention for STEMI: A Review of "*Ideal*" vs. "*Real-world*" Clinical Scenarios

**DOI:** 10.31083/RCM47494

**Published:** 2026-03-17

**Authors:** Chun Shing Kwok, Rahul Potluri, Jaydeep Sarma, Kevin R Bainey, Josip Andelo Borovac

**Affiliations:** ^1^Department of Cardiology, Mid Cheshire Hospitals NHS Foundation Trust, CW1 4QJ Crewe, UK; ^2^Birmingham City University, B5 5JU Birmingham, UK; ^3^Department of Cardiology, Royal Devon and Exeter NHS Trust, EX2 5DW Exeter, UK; ^4^Department of Cardiology, Manchester University NHS Foundation Trust, M13 9WL Manchester, UK; ^5^Mazankowski Alberta Heart Institute and University of Alberta, Edmonton, AB T6G 0M9, Canada; ^6^Division of Interventional Cardiology, Department of Ischemic Heart Disease & Department of Cardiovascular Diseases, University Hospital of Split (KBC Split), 21000 Split, Croatia; ^7^Department of Pathophysiology, University of Split School of Medicine, 21000 Split, Croatia

**Keywords:** ST-elevation myocardial infarction, percutaneous coronary intervention, patient pathways, diagnosis, delay, misdiagnosis, outcomes, delayed diagnosis

## Abstract

Care processes and outcomes for patients undergoing primary percutaneous coronary intervention (PCI) for ST-segment elevation myocardial infarction (STEMI) remain heterogeneous. A “patient pathway” framework—defined as the sequence of clinically relevant events from symptom onset through diagnosis, reperfusion, and early recovery—can help identify real-world points of failure and opportunities for system-level improvement. In this narrative review, we contrast an “ideal” STEMI pathway with the pathways commonly observed in routine practice for patients treated with primary PCI, and we contextualize deviations from best practice from patient, clinician, health service, and societal perspectives. From the patient's perspective, the priority is rapid symptom recognition and seeking care; however, delays are frequent, particularly in individuals with mild, atypical, or non-classical presentations, prolonging total ischemic time and increasing myocardial injury. Clinicians aim to diagnose STEMI promptly and initiate evidence-based therapy and reperfusion without delay, yet diagnostic uncertainty and competing differentials can contribute to missed or late diagnoses. Health systems seek to provide timely, efficient, and cost-effective emergency revascularization, but performance is influenced by pre-hospital logistics, triage, catheterization laboratory availability, and inter-hospital transfer processes. At the societal level, STEMI imposes substantial mortality, morbidity, and economic burden through premature death and disability. We synthesize evidence on delays to revascularization, misdiagnosis, populations at risk for atypical presentation, and pragmatic interventions to improve care. We conclude that pathway-based analyses offer a structured approach to defining desirable STEMI care trajectories and to reducing missed opportunities for better outcomes.

## 1. Introduction

ST-elevation myocardial infarction (STEMI) is a significant cause of worldwide mortality in adults [1]. The American and European guidelines recommend emergency coronary revascularization, where primary percutaneous coronary intervention (PCI) is the preferred management of STEMI [2, 3]. However, there is considerable variability in treatment and outcomes among patients, as survivors may return to near normal daily living, while other patients may be affected by heart failure.

The factors that contribute to the receipt of primary PCI and associated patient outcomes can be divided into those that relate to the pre-PCI space, time of PCI, and time post-procedure. The pre-PCI delay is complex, as it can be related to patients failing to recognize the significance of their symptoms, patients seeking professional advice from individuals who lack expertise in recognizing acute coronary syndrome, misdiagnosis by healthcare professionals, and the availability of care in the area where patients live, leading to delayed PCI. At the time of the PCI, decision-making can affect outcomes such as decisions about stenting. For example, metal stents require antithrombotic therapy, which can place patients at risk, as well as the risk of access site complications, and inadequate stent or lesion optimization, which leads to the potential risk of secondary short- or long-term complications. Post procedure focus is on preventing complications related to acute ischemia and other adverse events, such as secondary ischemic events and bleeding from antithrombotic use. These factors contribute to the complexities of real-world practices, but their implications are not often considered in decision-making about clinical practices and research. The patient pathway review was recently developed to systematically evaluate these real-world complexities, which contribute to different patient pathways in care and outcomes [4]. This has been used to explore what happens to patients who have stable chest pain [5] and atrial fibrillation [6]. This approach has not been applied before in the context of patients with acute STEMI who undergo primary PCI. In this report, we review the patient pathway of patients undergoing primary PCI for STEMI.

## 2. Pathway Construction

### 2.1 Starting Point (Baseline)

The starting point of the patient pathway review is the onset of STEMI in the community. It is notable that STEMI can also develop among hospitalized patients, but these patients are more likely to have accessed professional input and evaluation compared to those in the community. In addition, the focus of this work is on patients where there is access to primary PCI as opposed to patients living in geographic areas where there are protocols for fibrinolysis.

### 2.2 “Ideal” Patient Pathway Construction

The ideal patient pathway is defined as a sequence of clinically relevant events. This is based on the most desirable clinical activities for a patient, which considers the onset of symptoms, presentation to the healthcare professional, evaluation and investigations, diagnosis, management, and response. It is also what most professionals who look after patients would agree represents the most common scenario for patients with STEMI who receive primary PCI.

### 2.3 “Real-world” Patient Pathway Construction

The real-world patient pathway systematically considers all the stages identified in the ideal patient pathway. The systematic approach refers to the evaluation of each stage of the ideal patient pathway for potential deviations without ignoring certain stages. In this step of the evaluation, there is no assumption that patients know about symptoms and the need to present to professionals, to ensure they do not bias the pathway by presenting to these individuals. Similarly, there will be no assumption that the diagnosis made by clinicians will be correct. Based on clinical experience, potential adverse events will also be considered.

### 2.4 Explaining the “Real-world” Pathway

A tenet of the patient pathway review is that events happen for certain reasons. In order to understand why events occurred, they may be rationalized by considering different perspectives. The perspectives considered are the patient perspective, clinician perspective, health service perspective, and societal perspective.

## 3. The “Ideal” Patient Pathway for STEMI

**Fig. 1.**
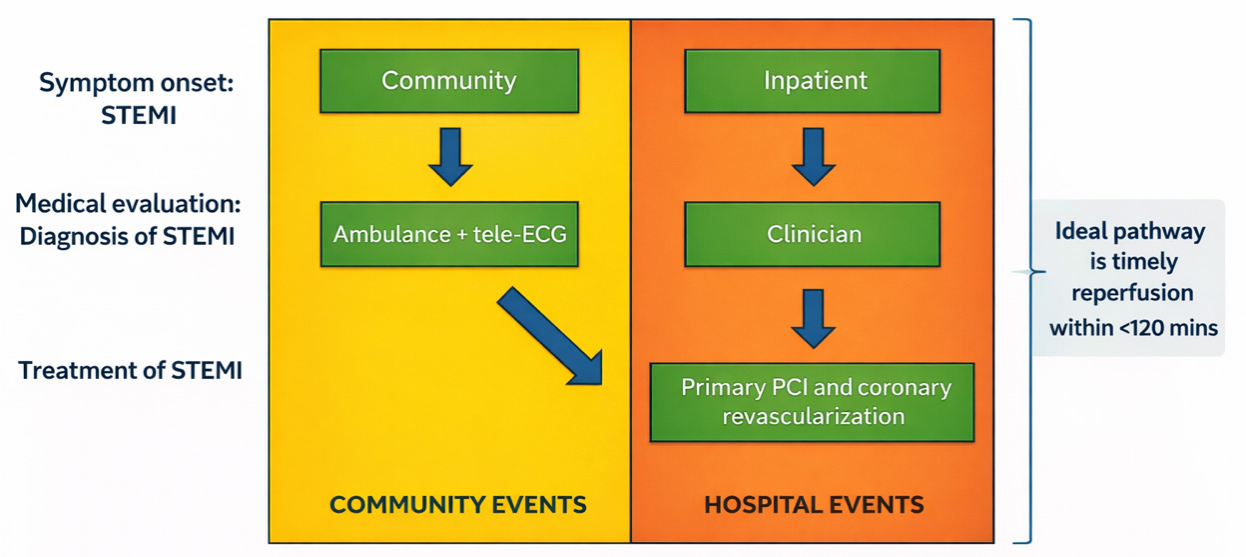
**The *“ideal”* care pathway for patients with STEMI**. STEMI, ST-segment elevation myocardial infarction; ECG, electrocardiogram; PCI, percutaneous coronary intervention.

The “ideal” patient pathway is shown in Fig. 1. From the starting point of the onset of STEMI, patients would typically develop chest pain either in the community or in the hospital. The patient would recognize their symptom(s) as an acute problem that requires urgent medical attention. As STEMI is an emergency, a patient in the community would ideally call an ambulance to take them to the hospital. The ambulance may have tele-electrocardiogram (ECG) capabilities so that the ECG can be reviewed by specialists. The hospital clinicians would identify that the patient has a STEMI and they would enter a treatment pathway that involves emergency primary PCI. This pre-hospital activation from the ambulance would prompt activation of the cardiac catheterization laboratory, reducing ischemic time [7]. The ideal time from onset of symptoms/chest pain to reperfusion would be less than 120 minutes. The patient would then be observed for a few days before discharge without short- or long-term complications.

## 4. The “Real-world” Patient Pathway

**Fig. 2.**
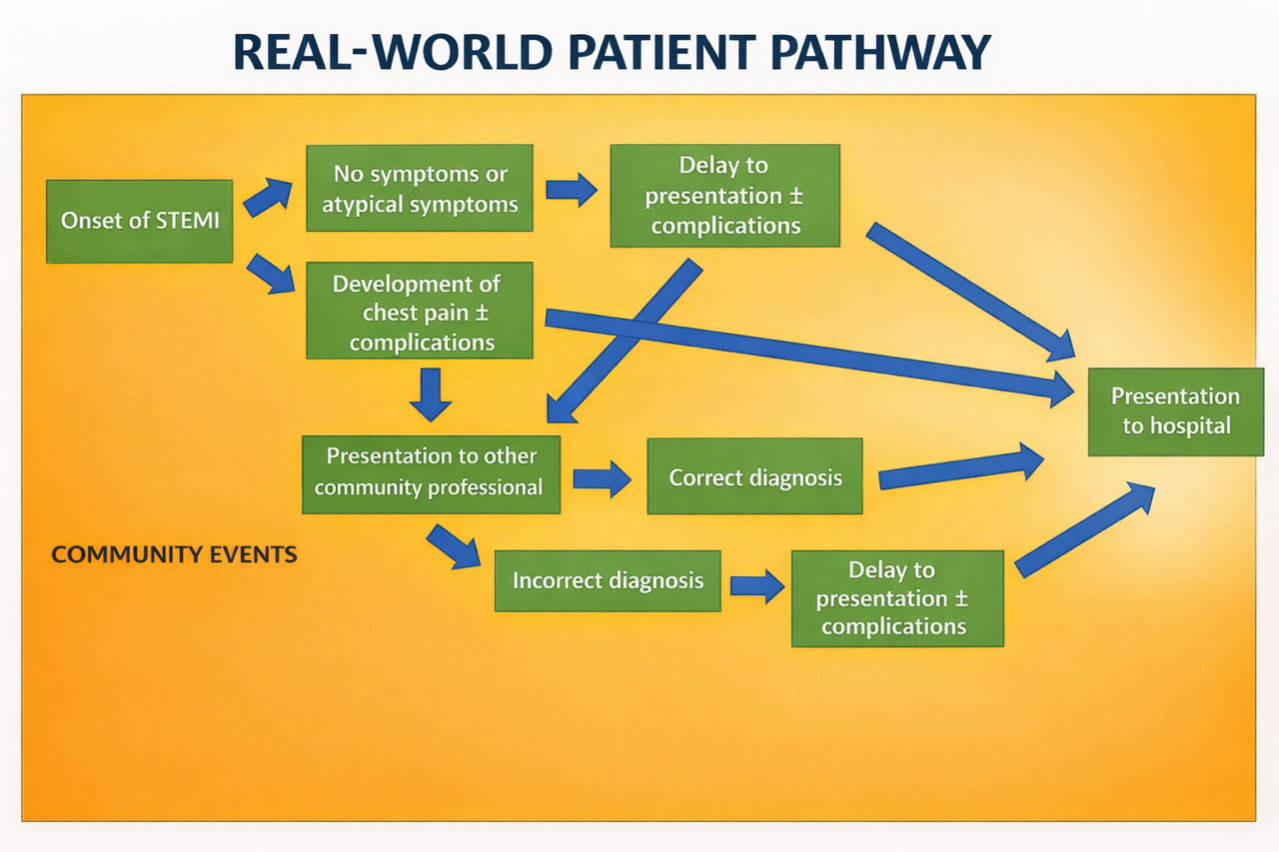
**A *“real-world”* care pathway in the community for patients with STEMI**.

**Fig. 3.**
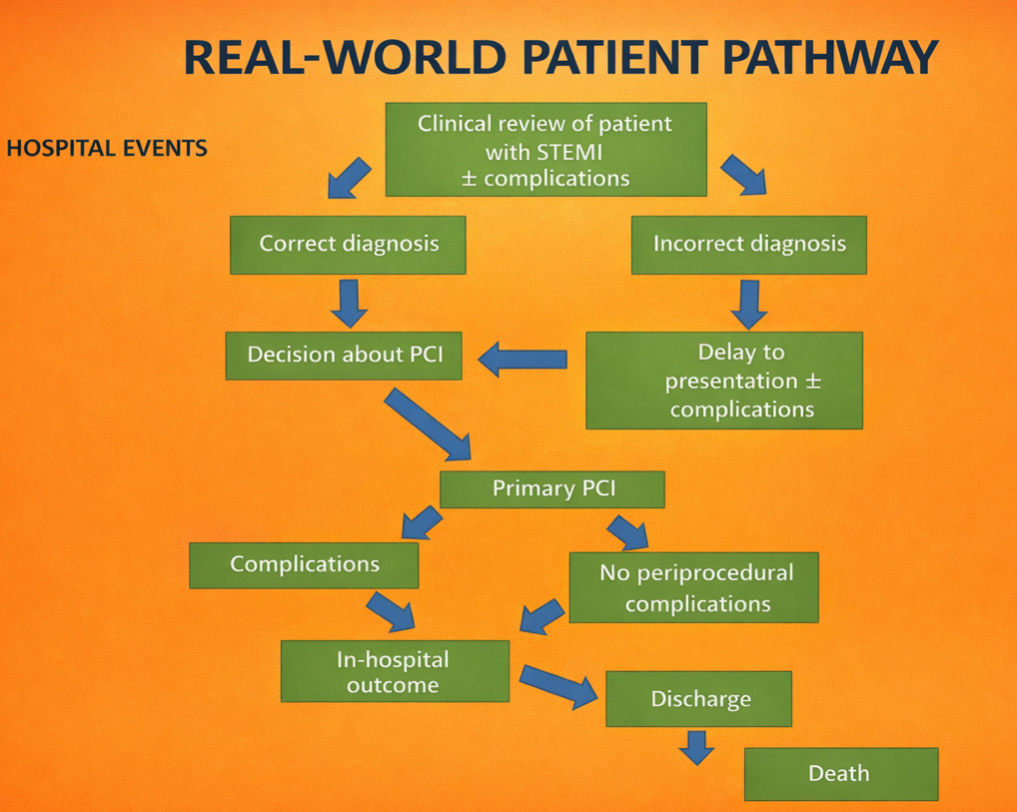
**A *“real-world”* care pathway in a hospital for patients with STEMI**.

The “real-world” pathway expands on the events in the community as well as those in the hospital, as shown in Figs. 2,3, respectively. From the onset of STEMI, patients will most commonly have chest pain. However, it is well recognized in the literature that some patients may have no chest pain or atypical symptoms, including nausea, sweating, palpitations, fatigue, and lightheadedness [8], and an atypical presentation is more common in elderly patients, female patients, and patients with diabetes mellitus [9]. There have even been reported cases of STEMI with no symptoms [10], and such patients with silent myocardial infarction may present with complications of ischemia, such as heart failure or syncope due to cardiac arrhythmia and sudden death. Some patients with severe coronary or occlusive coronary disease may have complications with their STEMI, such as acute pulmonary edema. The challenge is that patients may have a variety of symptoms and not all will present to the hospital where definitive treatment is available. Some patients may not be aware that their symptoms present a life-threatening emergency and see a community practitioner, such as a family doctor, general practitioner, or specialist in outpatient settings. While patients with classic symptoms of STEMI, including chest tightness radiating to the jaw or left arm, would likely be identified by clinicians, it is less likely that some cases of STEMI, especially those with mild symptoms, may be correctly diagnosed. There is literature to suggest that patients may be wrongly diagnosed with non-specific chest pain, gastrointestinal disease, musculoskeletal pain, and arrhythmias [11]. The availability of tests and the experience of clinicians have an important role in influencing whether the correct diagnosis is made. In particular, not all family doctors or general practitioners will have access to ECGs, and there is a further need for clinicians to accurately be able to interpret ECGs. The real-world setting is also complicated by the availability of community health services in the area where the patient lives, as well as other factors such as whether the healthcare is government-funded or privately insured. The key consideration in the real-world pathway leading up to hospitalization is the patient’s recognition of the problem, the choice of professional they choose to seek help from and the skill of the clinician to identify STEMI.

Most hospitals are prepared to identify STEMI as an acute myocardial infarction, and many are able to treat or have algorithms to urgently revascularize patients. In the hospital environment, the first step is assessing whether the patient may or may not be identified as STEMI. In the current practice where ECGs are available, the only issues preventing diagnosis of chest pain are the request for an ECG and the interpretation of the findings. However, as highlighted earlier, patients may have atypical symptoms in the context of STEMI. In cases where there are delays due to complications such as structural problems or acute heart failure, these affect decisions about PCI. Decisions about PCI are frequently based on ECG findings and symptoms, as ongoing chest pain with electrocardiographic changes prompts emergency revascularization even if the presentation is delayed. The case is less certain for patients with long delays to presentation, Q-waves on ECG, and who have become pain-free, as it is arguable that revascularization may not be of benefit unless the myocardium is identified as viable. Nevertheless, in most cases of STEMI, patients will undergo primary PCI. This is the case particularly for patients who present to hospitals with primary PCI capabilities, but most hospitals lacking PCI facilities have protocols to facilitate transfer to a hospital that does offer these services. In some cases, decisions may be made about the use of fibrinolysis in the context of a pharmacoinvasive strategy when timely PCI cannot be performed [12]. The primary PCI procedure is not without recognized risks, including stroke, vascular injury, major bleeding, including coronary perforation, slow or no flow, arrhythmias, and contrast nephropathy. The patients can also go into cardiogenic shock or arrest during the procedure. Decision making periprocedurally, including access site choice, antithrombotic choice, decision to stent versus balloon angioplasty, use of intracoronary imaging and devices such as intra-aortic balloon pump, and stringent or liberal use of contrast, can affect periprocedural complications. In most cases, the procedure will be carried out without complications, with most patients having a hospital outcome of survival to discharge.

The patient pathway also applies post-discharge after STEMI. Once discharged, patients may be free of complications or develop longer-term complications such as major bleeding from antithrombotics, stent thrombosis, in-stent restenosis, or heart failure. In the acute and subacute period, adherence to dual antiplatelet therapy is of particular importance. Non-adherence to antiplatelet therapy is associated with critical cardiac events post PCI [13]. Cardiac rehabilitation is also recommended to manage risk factors, and failure to enroll in cardiac rehabilitation may result in secondary cardiovascular events [14]. There may also be missed opportunities for secondary prevention, which includes medications and lifestyle changes. Risk factors that can be managed include elevated blood pressure, diabetes mellitus identification, and smoking cessation. Promotion of a healthier lifestyle may include the adoption of a healthier diet, weight loss among obese patients, and promotion of regular exercise. The way care is delivered may be specific to the setting, as reviews post-discharge and the timing of follow-ups may be with the hospital or community teams.

## 5. Explaining the “Real-world” Pathway

### 5.1 The Patient Perspective

From the patient’s perspective, most patients with STEMI are symptomatic, but what patients experience may vary considerably. The classic presentation of STEMI is chest pain or tightness radiating to the left arm or jaw associated with nausea and diaphoresis. Other patients may be asymptomatic, and some may experience symptoms related to complications such as respiratory distress in acute pulmonary edema, or syncope with cardiac arrest. The key consideration is how patients interpret their symptoms and their behaviors in response to these symptoms. Patients may not be aware that they are having a heart attack, so they may not seek the right professional who can diagnose the condition. This contributes to heterogeneity in how patients present. They may seek to include a community practitioner such as a family physician or general practitioner, an outpatient cardiologist, self-present to emergency departments, or call an ambulance. As a point of first contact, the patient would desire to know what the problem is and how to alleviate symptoms.

The willingness and decision about who to seek medical help from is complex and can even be influenced by medical knowledge, geography, and the local healthcare system. Medical knowledge can influence propensity to seek help. While patients living in urban areas will likely have access to both community and hospital services, those living in rural areas may only have community services available for first contact. If there is a potential benefit associated with coronary revascularization, this is likely their preference even if there are associated risks. This includes both primary PCI and intravenous thrombolysis. The healthcare system, whether public or private, may also have an influence on patients’ willingness to seek help, as the cost of healthcare in private healthcare systems may deter patients from seeking help because of the potential financial burden. In summary, the main priorities of the patient are to avoid long-term harm in the form of complications and alleviate symptoms, but the patient’s actions can affect their care pathway, as there may be a delay related to seeking help, and their choice of healthcare professional to seek help from.

### 5.2 The Clinician Perspective

The aim of the clinician caring for a patient with STEMI is to promptly diagnose the condition and instigate the emergency treatment to prevent any further harm. The clinicians’ perspective is therefore divided into the clinicians who make the diagnosis and those who deliver the primary PCI treatment.

STEMI is a recognized emergency that can lead to ventricular arrhythmias and cardiac arrest. Delays to diagnosis and misdiagnosis can have serious and life-threatening consequences. Usually, it is not a diagnostic challenge for clinicians of patients who present with typical symptoms. However, some patients may not have chest pain or have non-specific symptoms such as nausea and vomiting. The key to accurate diagnosis is the ECG, and all paramedics are trained to take an ECG from patients. A problem with patients being reviewed in the community is that there may not be immediate access to an ECG, especially in rural areas. Most patients coming to the hospital will have an ECG performed, and failure to interpret an ECG correctly can be a problem. It is recognized that the misinterpretation of the ECG is a major contributor to the missed diagnosis of myocardial infarction. Once the diagnosis is made, there are usually pathways to either treat with fibrinolysis or consider transfer to a nearby center for emergency coronary revascularization. These decisions can be made in a pre-hospital setting with advancements in STEMI networks.

The second major consideration from a clinician’s perspective is that of the primary PCI operator. The success of the procedure is influenced by the time to procedure, as the benefit of coronary revascularization is reduced with prolonged time from the onset of ischemia. In addition, the aim of primary PCI treatment is to prevent long-term harm, but the procedure itself is not without risk. Use of a coronary stent requires a duration of antiplatelet therapy, which can cause bleeding. The implanted stents can also acutely thrombose or chronically stenose over time. Complications such as embolic stroke, coronary perforation, aortic or coronary dissection, and reperfusion injury (no reflow phenomenon) can occur in the periprocedural period. Patients with a delayed presentation may no longer benefit from revascularization, so these patients may not even go to the catheter laboratory. Decisions may be made not to go to the catheter laboratory, and these patients are managed medically. In addition, any complications or sequelae of the STEMI can also make the primary PCI procedure more difficult. For example, if patients go into acute pulmonary edema due to left ventricular failure or develop life-threatening arrhythmias such as bradycardia or ventricular arrhythmia, the procedure can be more challenging. Therefore, from a PCI operator’s perspective, delay to treatment should be minimized, and where possible, patients should be optimized prior to intervention. In addition, once the procedure is over, secondary prevention measures should be instigated, which include antiplatelet drugs, statins, beta-blockers, angiotensin converting enzyme or angiotensin receptor blocker, and anticoagulation if indicated. The decisions made regarding patient care from the moment they go into the laboratory for possible intervention are governed by the experience of the operator and their approach to minimizing risks to patients.

### 5.3 Health Service Perspective

From a healthcare service perspective, the ability to deliver acute reperfusion therapy is the gold standard treatment for STEMI. The consequences of missing this treatment can be significant, as patients can have out-of-hospital cardiac arrest, leading to mortality in the community. If the patient survives the acute ischemia, they may develop heart failure with a variable degree of severity, with some patients being able to return to independent living while other patients lose independence as a result of disabling symptomatic heart failure. Timely treatment can therefore have major implications on the individual and on society, in relation to work productivity for those who are still in employment at the time of the coronary event and their ongoing care needs. However, it is impossible geographically to enable all patients with acute STEMI to have timely primary PCI, considering individuals who live in rural areas. Nevertheless, these patients should have pathways for treatment, as acute myocardial infarction is common, and these include fibrinolysis as a bridge to a PCI-capable hospital (pharmacoinvasive strategy) or direct transport from the community to a PCI center, provided timely metrics are achieved. However, the long delays to accessing primary PCI are increasingly common, putting patients at risk, resulting in increased burden to the healthcare system acutely and in the long term (i.e., recurrent heart failure). In fact, from a health service perspective in a ‘real-world’ STEMI scenarios in Norway, in the areas where primary PCI could not be achieved in a timely fashion (due to geographic constraints), a pharmacoinvasive strategy proved provide greater benefits compared to delayed primary PCI, improving survival but with the offset of more bleeding events [15]. From a healthcare perspective, options for a dual reperfusion strategy should be considered to ensure patients receive the best reperfusion therapy to reduce ischemic time and clinical events. While it is expected that patients with chest pain should know to seek help from healthcare professionals and those who assess them should consider the diagnosis of acute STEMI, the case may not be the same for patients with atypical symptoms, such as those without chest pain. Because of the timely nature of the treatment of STEMI, most health services will have treatment pathways for emergency revascularization to minimize delay to treatment should a patient be identified to have STEMI.

### 5.4 Societal Perspective

The impact of STEMI on the population is significant. It is responsible for a high proportion of sudden cardiac deaths in the community. Therefore, most health services have care pathways in place to manage patients with STEMI because of its life-threatening nature. It is expected that in most urban centers, there are interventional cardiologists who can provide primary PCI treatment 24 hours a day and 7 days a week. However, in rural areas, there may be mechanisms to transport patients urgently, either by ambulance or helicopter, to PCI centers for treatment or care pathways that include fibrinolysis within the context of a pharmacoinvasive strategy.

Coronary artery disease due to atherosclerosis is not always predictable. Some patients develop soft plaque or hard calcific plaque, which may or may not be stenotic. Unfortunately, coronary occlusion may occur acutely in STEMI patients due to plaque rupture in coronary vessels with normal flow pre-plaque rupture, so there may be no opportunity to stent the lesion prior to STEMI. This is different from patients who have chronic angina due to stenotic coronary disease and develop acute coronary artery occlusion in vessels that are previously narrowed. In the latter case, it would be safer to treat patients with either medical therapy to delay plaque progression or stenting electively as a day case prior to acute occlusion.

There are many established risk factors for cardiovascular disease, such as obesity, hypertension, smoking, and family history of premature coronary disease. From a societal perspective, it would be more desirable to manage these risk factors than for patients to develop STEMI. Health promotion can have an important role in this, which includes measures to increase physical activity, weight loss among the obese, smoking cessation, and a healthier diet.

The cost and societal impact of STEMI can be significant. Cardiac arrest and death are an unfortunate possible outcome for patients, which means the loss of a person, family member, and a contributor to society. In addition, ischemic cardiomyopathy can develop, resulting in disabling symptoms, loss of productivity, physical disability, and loss of independence, which can also have a psychological impact, precipitating depression. Therefore, optimal pathway management should be promoted, maximizing the chance of survival and independence.

## 6. Discussion

Consideration of patient pathways provides an opportunity to learn from real-world activities and associated outcomes for patients. Of particular interest are pathways for patients that result in avoidable harm. In the patient pathway, the first key decision is that the patient recognizes that there is something wrong, as it is impossible to help patients if they do not present to appropriate healthcare professionals. To reduce the harm associated with a delay in seeking help, public health education can be used to promote the importance of seeking help for chest pain and an example of this is the first of the “Help Us Help You” campaign in the United Kingdom designed to encourage people to call an ambulance for suspected chest pain and tackle myths about heart attacks [16]. The second key consideration is reducing misdiagnosis when patients present to healthcare professionals. Unfortunately, not all professionals are skilled at reading ECGs, and experience with assessing patients with acute STEMI, inaccurate ECG interpretation, and diagnostic uncertainty or confusion have been reported to be linked to missed acute myocardial infarction [17]. It is important to educate those who are assessing patients on the frontline, whether they are doctors, nurses, or paramedics, about the possible symptomatology of patients with acute coronary syndrome and STEMI recognition on ECG. This is important as chest pain has multiple potential causes, such as gastritis, pulmonary embolism, aortic dissection, musculoskeletal pain, and chest infection.

Data from the American Heart Association Get With the Guidelines-Coronary Artery Disease registry from 2020 to 2022 provides some insight into the real-world variation in care for patients with STEMI or STEMI equivalents [18]. The analysis of 73,826 patients from 503 US hospitals found that 59.5% of patients admitted directly to PCI-capable hospitals achieved a first medical contact-to-device time of 90 minutes or less, and 50.3% of transferred patients had a first medical contact-to-device time of 120 minutes or less. This evaluation found that failure to meet the time targets was associated with a 2.21-fold increase in odds of mortality. The time from the first medical contact-to-device in patients with STEMI should be reduced where possible, but there may be complex factors that are institution-specific, such as the demographics of the catchment population and the geographical area of coverage, which might influence this metric.

Misdiagnosis is an everyday reality. Data from the Korean Acute Myocardial Infarction Registry suggested that 1.4% of patients with a final diagnosis of STEMI are misdiagnosed, and these patients have a 5 times longer door-to-angiography duration and a 1.8-fold increase in risk of 1 year mortality [19]. A review of 15 studies evaluating misdiagnosis in acute myocardial infarction suggests a rate of between 1–2%, whilst another study suggested no difference in 30-day and 1-year mortality [20]. In addition, the rate of false-positive STEMI activations is a real-world challenge. An Australian study of 1736 STEMI cases found that false positive STEMI activation was 2.75% among pre-hospital activations, 5.4% among emergency department activations, and 6% in in-hospital transfer activations [21]. There may be opportunities to apply artificial intelligence (AI) to ECG analysis in order to improve care of patients with STEMI, as an analysis of 1032 patients with suspected STEMI found that an AI ECG model outperformed standard triage with greater sensitivity (92.0% vs 71.0%) and reduced false STEMI activation (7.9% vs 41.8%) [22].

There are some groups of patients who have a tendency to present atypically with STEMI, which contributes to patient delay, misdiagnosis, and suboptimal care. One study found that women were less likely to report chest pain than men, and they were more likely to complain of nausea, palpitations, dyspnea, fainting, and back pain [23]. Another evaluation of 550 patients who presented to the emergency department with coronary heart disease found that women were more likely to present with nausea and/or vomiting and indigestion [24]. Compared to younger patients, elderly patients with myocardial infarction are more likely to present with dyspnea, fatigue, and symptoms of heart failure, compared to typical chest pain [25]. It has been suggested that the atypical presentation of elderly patients with STEMI may be related to changes in left ventricular pressure during ischemia, acute left ventricular systolic dysfunction, age-related pulmonary changes, comorbid conditions, altered pain perception, ischemic preconditioning, acute reductions in central nervous system blood supply, and an inability to recall or report symptoms [26]. A study of 4450 patients with diabetes found that these patients are less likely to present with typical chest pain, which contributes to treatment delay among patients suffering from acute myocardial infarction [27]. In addition, patients with diabetes have attenuated symptoms, which often lead to a delay in seeking attention, which has a downstream negative consequence in the timeliness of treatment [28]. Therefore, targeted interventions are needed for high-risk patients and clinician education to overcome diagnostic bias in patients with STEMI.

This work focuses on pathways for STEMI, but there has been a recent paradigm shift to occlusive myocardial infarction [29]. A study in emergency care settings found that patients without ST elevation who had occlusive coronary disease had significant delays in treatment compared to patients with ST elevation on ECG [30]. It is now recommended that, in the context of acute chest pain, STEMI based on ECG will miss a significant minority of patients with acute coronary occlusion [31]. STEMI is used in clinical practice because ECGs can be done in any clinical setting, including the community. The only way to confirm occlusive coronary disease is with computerized tomography coronary angiography (CTCA) or invasive angiogram, and not all patients undergo this evaluation. The focus on ECGs in the diagnosis of STEMI is important with increasing use of technology in cardiology, as AI-enabled ECG analysis has been shown to improve diagnostic accuracy and reduce false STEMI activations in the United States [32].

The patient pathway framework considers delays and system performance, and this systematic approach may help to identify areas of potential improvement in the care of patients with STEMI. In the pre-hospital phase, ECG transmission in STEMI patients, coupled with a system of care, reduces door-to-device times, first-medical-contact-to-device times, and mortality [33]. Once patients present to the hospital, AI-based triage system has also been shown to reduce the door-to-balloon time in the emergency department, which can help minimize preventable delays in patients with STEMI who undergo primary PCI [34]. In the post-discharge phase, patients who are revascularized may have left ventricular dysfunction, and some may benefit from wearable cardiac devices to prevent sudden cardiac death [35]. There are also healthcare-wide systems that can impact outcomes, as clinical guidelines also recommend the development of STEMI networks at community, regional, and national levels to ideally facilitate primary PCI to all patients with STEMI [36]. For example, the Mayo Clinic STEMI protocol has been shown to optimize the timeliness of reperfusion therapy by coordinated systems of care across 28 regional hospitals without PCI capabilities located up to 150 miles away across 3 states [37]. These potential areas of improvement require evaluation on a local or regional level for their potential benefit, as they are not without the requirement of resources to implement.

## 7. Conclusion

In this patient care pathway review, we define a framework for the real-world heterogeneity of clinical activities and outcomes for patients who undergo primary PCI for STEMI. From the onset of symptoms in the community, patients may develop typical or atypical symptoms, which may contribute to a delay in presenting to healthcare professionals. Delays can also be introduced because clinicians can misdiagnose patients, particularly those with atypical symptoms. Patients who are identified to have STEMI will then be taken to a center for revascularization, and the ultimate outcome is death or survival with or without complications. The patient, clinician, health service, and societal perspectives all align in the aim to reduce harm associated with STEMI, which includes measures to reduce delays to symptom identification and misdiagnosis, ultimately preventing future harm after the acute event.
